# The Effect of Nirmatrelvir-Ritonavir on Short- and Long-term Adverse Outcomes From COVID-19 Among Patients With Kidney Disease: A Propensity Score-Matched Study: Open Forum Infect Dis. 2024 Dec 31;12(1):ofae756. doi: 10.1093/ofid/ofae756

**DOI:** 10.1093/ofid/ofaf480

**Published:** 2025-08-21

**Authors:** Stanley A Rubin

**Affiliations:** Department of Medicine, School of Medicine, David Geffen School of Medicine at UCLA, Los Angeles, California, 90095, USA

To the  Editor—The informative paper by Strohbehn et al. on the outcome of patients with chronic kidney disease, defined as an estimated glomerular filtration rate (eGFR) of <60 mL/min, leaves two questions unanswered. The first question relates to dosing. From the legend of Table 1, [*] 52% of patients received nirmatrelvir 300 mg/ritonavir 100 mg for 5 days, whereas 48% received nirmatrelvir 150 mg/ritonavir 100 mg twice daily for 5 days. Yet, the recommended dose for patients with an eGFR of <60 mL/min is the lower dose [1]. Why did 52% of patients receive higher than the recommended dosage? The second question leads to the effect in this (probably) unintended higher-dosage regimen. That is, were there significant differences in outcomes between the two dosages?

**Table 1.** Patient Characteristics Before and After Propensity Score Matching

	Before Propensity Score Matching			After Propensity Score Matching		
Characteristic	Antiviral-Treated (n = 1723)	Untreated Historical Comparators (n = 940)	Standard Mean Difference	Antiviral-Treated (n = 1095)	Untreated Historical Comparators (n = 584)	Standard Mean Difference
Female, no. (%)	1001 (58)	471 (50)	0.16	600 (55)	308 (53)	0.05
Baseline age, mean (SD), y	76 (11)	73 (13)	0.27	76 (11)	74 (13)	0.08
Comorbidities, no. (%)						
Hypertension	1553 (90)	759 (81)	0.24	954 (87)	496 (85)	0.02
Diabetes	683 (40)	441 (47)	0.15	467 (43)	278 (48)	<0.01
Coronary artery disease	835 (48)	462 (49)	0.01	547 (50)	294 (50)	0.02
Congestive heart failure	118 (7)	93 (10)	0.10	86 (8)	61 (10)	0.05
Chronic obstructive pulmonary disease	256 (15)	153 (16)	0.04	168 (15)	102 (17)	0.03
History of cancer	874 (51)	370 (39)	0.16	519 (47)	265 (45)	0.02
Chronic kidney disease group						
eGFR 45–59.9	1341 (78)	549 (58)	0.39	807 (74)	375 (64)	0.08
eGFR 30–44.9	312 (18)	217 (23)	0.12	221 (20)	132 (23)	<0.01
eGFR <30, not on dialysis	15 (2)	74 (8)	0.26	15 (1)	28 (5)	0.09
Kidney failure	55 (3)	100 (11)	0.24	52 (5)	49 (8)	0.06
Medications, no. (%)						
Nonsteroidal anti-inflammatory drugs	303 (18)	175 (19)	0.03	205 (19)	117 (20)	0.01
Beta-blockers	706 (41)	381 (41)	<0.01	455 (42)	256 (44)	0.03
Statins	1088 (63)	436 (46)	0.34	641 (59)	315 (54)	0.02
Angiotensin-converting enzyme inhibitors/angiotensin II receptor blockers	918 (53)	359 (38)	0.31	521 (48)	262 (45)	0.04
Proton pump inhibitors	509 (30)	286 (30)	0.02	356 (33)	198 (34)	0.02
Diuretics	694 (40)	337 (36)	0.09	417 (38)	240 (41)	0.06
Immunosuppressants	97 (6)	47 (5)	0.03	62 (6)	29 (5)	0.02
Related health history, no. (%)						
Prior COVID-19 vaccination doses						
None	63 (4)	182 (19)	0.40	63 (6)	78 (13)	0.07
Primary series (1–2 doses)	224 (13)	460 (49)	0.72	224 (20)	227 (39)	0.03
3+ doses	1436 (83)	298 (32)	1.11	808 (74)	279 (48)	0.09
Time since last COVID-19 vaccination dose, no. (%)						
<180 d	907 (53)	407 (43)	0.19	760 (69)	299 (51)	0.05
≥180 d	816 (47)	533 (57)	0.19	335 (31)	285 (49)	0.05
Prior infection detected by PCR, no. (%)	26 (2)	27 (3)	0.08	22 (2)	15 (3)	<0.01
No. of serum creatinine measurements within 12 mo prior, no. (%)						
1 prior measurement	513 (30)	245 (26)	0.09	286 (26)	150 (26)	<0.01
2–4 prior measurements	785 (46)	350 (37)	0.17	492 (45)	226 (39)	0.06
5+ prior measurements	425 (25)	345 (37)	0.25	317 (29)	208 (36)	0.05
Hospitalizations within 12 mo prior, no. (%)						
0 prior hospitalizations	1505 (87)	708 (75)	0.28	922 (84)	458 (78)	0.05
1–2 prior hospitalizations	146 (9)	124 (13)	0.14	115 (11)	68 (12)	0.02
3+ prior hospitalizations	72 (4)	108 (11)	0.23	58 (5)	58 (10)	0.10

Among the matched nirmatrelvir–ritonavir-treated patients, 572 (52%) received nirmatrelvir 300 mg/ritonavir 100 mg twice daily for 5 days, and 523 (48%) received nirmatrelvir. One hundred fifty milligrams/ritonavir 100 mg twice daily for 5 days. Among matched comparators, 189 (32%) were infected when the delta variant was the most prevalent circulating variant (15 July 152021 to 24 December 2021) and 395 (68%) when Omicron was the most prevalent circulating variant (24 December 2021 to 15 March 2022).

Abbreviations: COVID-19, coronavirus disease 2019; eGFR, estimated glomerular filtration rate; PCR, polymerase chain reaction.

## Notes

***Financial support.*** No financial support.
